# EHMTI-0213. Does prior craniofacial nociception lead to an increase in cortical excitability?

**DOI:** 10.1186/1129-2377-15-S1-F25

**Published:** 2014-09-18

**Authors:** W Supronsinchai, A Srikiatkhachorn

**Affiliations:** 1 Department of Physiology Faculty of Dentistry, Chulalongkorn University, Bangkok, Thailand; 2 Department of Physiology Faculty of Medicine, Chulalongkorn University, Bangkok, Thailand

## Introduction

Attacks of migraine following the intense craniofacial somatosensory activation is observed clinically. The mechanism underlying this phenomenon may involve the trigeminal influence on cortical excitability. Whether or not this evoked attack is specific to craniofacial nociception is still unclear.

## Aims

To compare the effect of prior craniofacial and extra-craniofacial nociception on development of cortical spreading depression (CSD) and CSD-evoked cortical hyperemia.

## Methods

Formalin 10% (0.1 ml.) was subcutaneously injected into the forehead and forepaw of adult male Wistar rats (7 each) in order to induce nociception. Saline of the same volume was given to the control animals. One hour after injection, CSD was induced by application of 3 mg of solid potassium chloride on rat’s parietal cortex. The depolarization shift (DC shift) and CSD-evoked changes in cortical blood flow were recorded for one hour.

## Results

Nociceptive activation induced by formalin injection into forehead and forepaw significantly increased the development of CSD and CBF. The number of DC shifts was 12±2, and 15±1 in the control and facial nociception groups, respectively. Similar pattern was observed in the forepaw nociception group. The number of DC shifts was 12±2, and 17±2 in the control and forepaw nociception groups, respectively. Characteristics of CSD-evoked cortical hyperemia were not different comparing between facial and forepaw nociception groups.

## Conclusion

Our findings indicate that acute nociceptive activation can facilitate the development of CSD regardless of site of nociception. These results imply that nociception-evoked cortical hyperexcitability may involve modification of central modulating system and does not specific to trigeminal nociception.

No conflict of interest.

